# Bioactive Polyphenolic Compounds from Propolis of *Tetragonula carbonaria* in the Gibberagee Region, New South Wales, Australia

**DOI:** 10.3390/foods14060965

**Published:** 2025-03-12

**Authors:** Dylan W. Ebner, Damon C. Woods, Trong D. Tran

**Affiliations:** 1 Centre for Bioinnovation, University of the Sunshine Coast, Sippy Downs, QLD 4556, Australia; debner@usc.edu.au (D.W.E.); dwoods1@usc.edu.au (D.C.W.); 2 School of Science, Technology and Engineering, University of the Sunshine Coast, Sippy Downs, QLD 4556, Australia

**Keywords:** *Tetragonula carbonaria*, stingless bee propolis, flavonoids, polyphenolics, antioxidant, antidiabetic

## Abstract

Stingless bee propolis has emerged globally as a new source of bioactive molecules that can advance human health. However, limited research has been conducted on Australian stingless bee propolis. This study investigated the chemical composition and biological activity of the propolis produced by the stingless bees *Tetragonula carbonaria* from Gibberagee, a distinct region of New South Wales state in Australia. Using bioassay-guided fractionation, twelve compounds were isolated, including six A-ring methylated flavonoids. Nine of these compounds demonstrated strong scavenging activity against 2,2-diphenyl-1-picrylhydrazyl radicals, with five exhibiting greater potency than vitamin C. Chemical structures of seven additional minor flavonoids were determined through an intensive MS/MS data analysis. In silico screening of these 19 compounds revealed that all, except for gallic acid, displayed a higher binding affinity to α-glucosidase than the antidiabetic drug, voglibose. This study showed that the Gibberagee stingless bee propolis is a promising source for nutraceutical and cosmeceutical applications owing to its strong antioxidant and antidiabetic properties. The unique profile of A-ring C-methylated flavonoids potentially provides valuable insights into its botanical origin.

## 1. Introduction

Stingless bees are a large group of social bees [1]. To date, there are more than 500 known stingless bee species distributing in the tropical and subtropical regions including the Neotropical region of South America (approximately 391 species), the Indo-Malayan region of Asia (approximately 60 species), the Paleotropical region of Africa (approximately 50 species), and the Australasia region of Oceania (approximately 11 species) [1]. Unlike honeybees, which build their nests primarily or even solely out of wax [2], most stingless bees use propolis, which is a mixture of plant resin and beeswax, for nest construction [3]. Compared to other bee products, such as honey, pollen, and beeswax, propolis contains the highest concentration of specialised plant metabolites and has valuable pharmacological activities [4]. The stingless bee propolis has been used in traditional medicines in Mexico, Brazil, Argentina, India, and Vietnam as a remedy for improving health and treating diseases [5]. Modern research has confirmed the biological activities of stingless bee propolis, including antioxidant, antimicrobial, anti-inflammatory and anticancer activities, which have a strong linkage with the chemical compositions of the original plant resin [6]. The chemical composition of stingless bee propolis consists of mainly polyphenolic and terpenoid compounds, whose ratio varies depending on the propolis type [7]. Possessing potent and wide-spectrum biological activities and diverse chemical composition, the propolis of stingless bees is considered a good resource for functional food and nutraceutical ingredients and potential novel pharmaceutical candidates [6,7].

Australia has more than 1700 native bee species, of which 11 are stingless and belong to the genera *Tetragonula* and *Austroplebeia* [8]. The domestication of *Tetragonula carbonaria* (*T. carbonaria*) colonies began in the 1980s [9]*,* and previous propolis research has only focused on samples of this species collected in Queensland, Australia [5]. Massaro and her colleagues reported that propolis from *T. carbonaria* had an anti-inflammatory property with a distinct chemical profile compared to propolis from honeybee *Apis mellifera* [10]. This propolis extract was also found to relax porcine coronary arteries in an endothelial-independent manner [11]. Chemical investigation of the *T. carbonaria* propolis harvested in South East Queensland resulted in the isolation of six flavanones, including (2*S*)-cryptostrobin, (2*S*)-stroboponin, (2*S*)-cryptostrobin-7-methyl ether, (2*S*)-desmethoxymatteucinol, (2*S*)-pinostrobin, and (2*S*)-pinocembrin [12]. These compounds showed antimicrobial activity against the Gram-positive *Staphylococcus aureus,* with MIC values ranging from 6.9 to 182.2 µg/mL [12]. Two novel phloroglucinols were found in the *T. carbonaria* propolis [13]. Although their biological activity has not been reported, the identification of these two compounds demonstrated the potential of finding novel molecules from Australian native stingless bee propolis, which originates from unique botanical sources. More recently, a potent antioxidant meroterpene, tomentosenol A, was identified from the *T. carbonaria* propolis [14]. This compound showed significant antifibrotic potential via the inhibition of transforming growth factor-β1 (TGF-β1)-stimulated, NFF-myofibroblast differentiation and soluble collagen production [14].

In recent years, polyphenolic compounds have attracted significant interest as possible therapeutic remedies and disease prevention against oxidative stresses linked with many chronic diseases such as diabetes mellitus, cancer, neural degradation, and cardiovascular diseases [15,16]. With diabetes mellitus being one of the world’s leading global health issues, leading to an increase of 80% risk of mortality for those with the disease, the need for more diverse disease prevention and management has become increasingly urgent [17]. One of the common treatments for diabetes mellitus is to retard the absorption of glucose through the inhibition of the enzyme α-glucosidase. While common therapeutic drugs (including acarbose, miglitol, and voglibose) that strongly inhibit this enzyme are readily available, these drugs often come with side effects, including diarrhoea, abdominal distention, and nausea [18]. Polyphenols have also been shown in the previous literature to potentially inhibit α-glucosidase, suggesting that incorporating additional polyphenols into the diet may reduce the need for these drugs and the progression and risk of diabetes mellitus [19,20]. With the aim to investigate the bioactive composition of the Australian native stingless bee propolis, this paper reports the potential antioxidant and antidiabetic compounds of the *T. carbonaria* propolis collected in Gibberagee, the Northeast region of New South Wales, Australia. Bioassay-guided fractionation led to the isolation of 12 phenolic and flavonoid compounds. Molecular networking analysis further revealed the presence of other flavonoid compounds in this propolis extract. The antidiabetic activities of these compounds were then assessed via an in silico assay.

## 2. Materials and Methods

### 2.1. Solvents and Reagents

Solvents used for extraction (ethanol—EtOH), HPLC (acetonitrile—MeCN), and LC-MS (MeCN and water—H_2_O) analyses were purchased from Merck (Melbourne, Australia). Ultra-pure water used for HPLC analysis was from an in-house Milli-Q system. The reagents, 2,2-diphenyl-1-picrylhydrazyl (DPPH) and L-ascorbic acid; NMR solvents, including deuterated chloroform (CHCl_3_-*d*) and deuterated methanol (MeOH-*d*_4_); and formic acid were purchased from Sigma-Aldrich (Melbourne, Australia).

### 2.2. Sample Collection and Extraction

Raw propolis from stingless bees *T. carbonaria* was harvested in June 2022 and stored in darkness at 4 °C. The raw propolis was then frozen at −20 °C, and the sample was powdered by grinding manually. Fine propolis powder (0.5 g × 10) was mixed with 5 mL of 70% (*v*/*v*) ethanol solution, heated at 65 °C for 30 min and then sonicated in an ultrasonic bath for 5 min. The sample was placed in ice for 10 min before being centrifuged at 3600 rpm at 4 °C for 10 min. The supernatant was dried under a vacuum using a GeneVac EZ-2 evaporator (Genevac, Ipswich, UK) to obtain dry propolis extract. Dry extracts were then combined for compound isolation.

### 2.3. Isolation and Purification

The extract (392.9 mg) was fractionated using a C_18_ Synergi Fusion HPLC column (4 μm, 100 × 21.2 mm) at a flow rate of 10 mL/min. The mobile phase consisted of H_2_O (solvent A) and MeCN (solvent B), running for 50 min with a linear gradient starting at 5% B for 10 min and increasing to 100% B for 30 min and then running isocratic for another 10 min to give six fractions, A–F (8.0 min for each fraction A–E, and 10 min for fraction F), which were collected. Fractions B–E showed free radical scavenging activity. Fraction B was purified on a Synergi Fusion HPLC column (4 μm, 100 × 21.2 mm) at a flow rate of 10 mL/min with a linear gradient from 5% B to 25% B for 30 min to yield compound **1** (2.2 mg, t_R_ = 15 min, 0.56%). Fraction C was loaded on the same Synergi Fusion HPLC column at a flow rate of 10 mL/min with a linear gradient from 20% B to 50% B for 30 min to obtain compounds **2** (3.2 mg, t_R_ = 18 min, 0.81%) and **3** (1.2 mg, t_R_ = 22 min, 0.31%). Fraction D was purified on the Synergi Fusion HPLC column at a flow rate of 10 mL/min with a linear gradient from 25% B to 60% B for 30 min to yield compounds **4** (4.2 mg, t_R_ = 20 min, 1.1%) and **5** (1.6 mg, t_R_ = 25 min, 0.41%). Fraction E chromatogram was obtained on the Inertsil Diol column (5 μm, 250 × 20 mm) at a flow rate of 10 mL/min using 80% hexane/20% isopropanol (solvent A) and 100% hexane (solvent B) as a mobile phase. The HPLC purification was run for 60 min with a linear gradient starting from 0% A to 25% A for 10 min, increasing to 65% A for 30 min and then to 80% A for the next 10 min and rising to 100% in the last 10 min to yield compound **6** (1.1 mg, t_R_ = 31 min, 0.28%), compound **7** (4.1 mg, t_R_ = 38 min, 1.0%), compound **8** (1.9 mg, t_R_ = 45 min, 0.48%), compound **9** (0.8 mg, t_R_ = 46 min, 0.20%), compound **10** (3.7 mg, t_R_ = 47 min, 0.94%), compound **11** (0.7 mg, t_R_ = 56 min, 0.18%), and compound **12** (0.8 mg, t_R_ = 59 min, 0.20%).

### 2.4. Evaluation of Antioxidant Activity Using DPPH Free Radical Scavenging Assay

The DPPH free radical scavenging activity of the propolis extracts at different concentrations was evaluated using the DPPH assay as described previously [21]. Briefly, the DPPH solution was prepared on the day of measuring at a concentration of 100 µM in MeOH. The propolis extracts (200 μL) at different concentrations were added to 600 μL of DPPH solution in Eppendorf tubes. The mixtures were kept in the dark at room temperature for 20 min before being plated to a 96-well plate (200 µL/well) and measured at 518 nm using a Perkin Elmer Enspire microplate reader (Waltham, MA, USA). All evaluations were performed in triplicate. Gallic acid and MeOH were used as positive and negative controls.

The percentage of inhibition of the DPPH radical for each sample was normalised and calculated using the following formula:

$$\%\;Inhibition=\left[1-\frac{\left(A_{S}-A_{P}\right)}{\left(A_{B}-A_{P}\right)}\right]\times 100$$

where A_S_ is the absorbance of the sample, A_P_ is the absorbance of the positive control, and A_B_ is the absorbance of the blank sample (negative control).

An absolute IC_50_ curve for each compound was generated using GraphPad Prism 10 (GraphPad, Boston, MA, USA) with a 95% confidence interval. The IC_50_ values were determined as the concentration required to inhibit 50% of DPPH free radicals and reported as mean ± standard deviation.

### 2.5. NMR Analysis

The NMR spectra were acquired on a Bruker Ascend 400 spectrometer (Billerica, MA, USA) equipped with a 5 mm room temperature probe operating at 400 MHz for ^1^H NMR and 100 MHz for ^13^C NMR. All experiments were acquired in automation (temperature equilibration to 298 K, optimisation of lock parameters, gradient shimming, and setting of receiver gain). Compounds **1**–**12** were dissolved in CHCl_3_-*d* or MeOH-*d*_4_. The ^1^H and ^13^C spectra were referenced to the residual deuterated solvent peaks at *δ_H_* 7.26 and *δ_C_* 77.0 (CHCl_3_-*d*) and *δ_H_* 3.31 and *δ_C_* 49.0 (MeOH-*d*_4_).

### 2.6. LC-QTOF MS Analysis

All LC-MS analyses were performed using an analytical scale Agilent 1290 uHPLC system combined with an Agilent 6546 QTOF mass spectrometer (Santa Clara, CA, USA). Separations were performed at 35 °C on a Zorbax Eclipse Plus C_18_ column (50 × 2.1 mm, 1.8 μm particle size, 95 Å pore size) with a flow rate of 0.4 mL/min. The mobile phase consisted of H_2_O (solvent A), and MeCN (solvent B), both acidified with 0.1% formic acid. The samples were separated using a 15 min programme, which started at 2% B for 0.5 min, increased to 100% B for 9 min, kept at this level for the next 3 min, reduced to 2% B for 1 min, and re-equilibrated for 1.5 min. The injection volume was 2 μL. The mass spectrometer is equipped with an ESI source. Mass spectra were acquired in both positive and negative ionisation modes using gas temperature of 250 °C, a gas flow of 5 L/min, a capillary voltage of 4000 V, a nebuliser pressure of 30 PSI, a sheath gas heater of 400 °C, a sheath gas flow of 12 L/min, and a nozzle voltage of 1000 V. Chromatographic separation and mass spectrometry were controlled using the Mass Hunter software (B.10.00, Agilent Technologies, Santa Clara, CA, USA).

### 2.7. Molecular Networking Analysis

The MS and MS/MS data acquired by Agilent uHPLC-QTOF MS were converted to mzML format using MSConvert (version: 3.0.23244-bc8a3ad) as part of the ProteoWizard suite and uploaded to the MassIVE MS data repository (https://www.nature.com/articles/nbt.3597, accessed on 15 August 2024). The Global Natural Product Social Molecular Network (GNPS) was created using the online workflow (https://ccms-ucsd.github.io/GNPSDocumentation/, accessed on 15 August 2024) on the GNPS website (http://gnps.ucsd.edu, accessed on 15 August 2024) [22]. The data were filtered by removing all MS/MS fragment ions within +/− 17 Da of the precursor *m*/*z*. MS/MS spectra were window-filtered by choosing only the top 6 fragment ions in the +/− 50 Da window throughout the spectrum. The precursor ion mass tolerance was set to 2.0 Da, and an MS/MS fragment ion tolerance of 0.5 Da was set. A network was created where edges were filtered to have a cosine score above 0.7 and more than four matched peaks. Further, edges between two nodes were kept in the network if and only if each of the nodes appeared in each other’s respective top ten most similar nodes. Finally, the maximum size of a molecular family was set to 100, and the lowest-scoring edges were removed from molecular families until the molecular family size was below this threshold. The spectra in the network were then searched against GNPS spectral libraries. The library spectra were filtered in the same manner as the input data. All matches kept between network spectra and library spectra were required to have a score above 0.7 and at least four matched peaks.

The GNPS output was then visualised using Cytoscape (version 3.10.2). The resulting retention times and precursor mass (+) *m*/*z* and (−) *m*/*z* from SIRIUS and GNPS outputs were matched to identify previously identified compounds through NMR analysis. Unknown compounds were then further identified using the differences in precursor mass and their MS/MS relationship with the neighbouring compounds. The Cytoscape data were then adapted for use in Biorender (version 2025). The GNPS original data can be accessed through the Supplementary Materials.

The SIRIUS software (version 5.8.6) was downloaded from the Lehrstuhl Bioinformatik Jena website (https://bio.informatik.uni-jena.de/software/sirius/, accessed on 1 December 2024). The MS and MS/MS data acquired by uHPLC-QTOF MS (6546 Agilent) was converted to mzML format using MSConvert (version: 3.0.23244-bc8a3ad) as part of the ProteoWizard suite. The mzML files contained the *m*/*z* of each compound and its relative intensity (%) extracted directly from the uHPLC-QTOF MS (6546 Agilent) using the Agilent MassHunter Workstation Software version B.08.00. To compute the molecular formulas, instrument type was set as Q-TOF; mass accuracy was set as 10 ppm; possible ionisation for positive mode was selected as [M+H]+, [M+K]+, and [M+Na]+; and possible ionisation for negative mode was selected as [M−H]−, [M−K]− and [M−Na]−. C, H, and O were selected for element searches, and the number of candidates was set to 10. Database formulas used were CHEBI, COCONUT, GNPS, KEGG, KEGG Mine, KnaPSnaCK, KnaPSnaCK, Maconda, Natural Products, PlantsCYC, PUBCHEM, and PUBMED. Structure elucidation by CSI: FingerID was set to search using the same adducts as SIRIUS and the same database sets. Canopus Class Prediction was also enabled.

### 2.8. In Silico Screening of Compounds Against α-Glucosidase

The Protein Data Bank structure of *α*-glucosidase from *Saccharomyces cerevisiae* (PDB code: 2ZQ0) was downloaded from the Research Collaboratory for Structural Bioinformatics (RCSB) Protein Data Bank (http://www.rcsb.org, accessed on 15 December 2024). The protein structure was analysed using Discovery Studio Visualiser 4.5, and its dimer was removed. Using galaxy.org.au, water molecules, heteroatoms, and ligands were removed, and polar hydrogen atoms were added (pH 7.4) to the structures as described by the official tutorials. The proteins’ binding and active site residues were determined as previously described by Uddin et al. [23] and then the binding site sphere was defined accordingly. The dimension of the sphere was 20 Å, the sphere’s centre (x, y, z) was (27.776172, 56.165741, 35.362259), and the exhaustiveness value was adjusted to 24. Binding affinity was calculated using galaxy.org.au [24]. The top 9 binding poses were opted for prediction, and results were analysed using Discovery Studio Visualiser 4.5. The docking study was validated by redocking and superimposing known *α*-glucosidase inhibitors (acarbose and vogibose) with the extracted protein from the crystal structure.

## 3. Results and Discussion

### 3.1. Antioxidant Activity of the Gibberagee Propolis and the Pure Polyphenols

**Figure 1:**
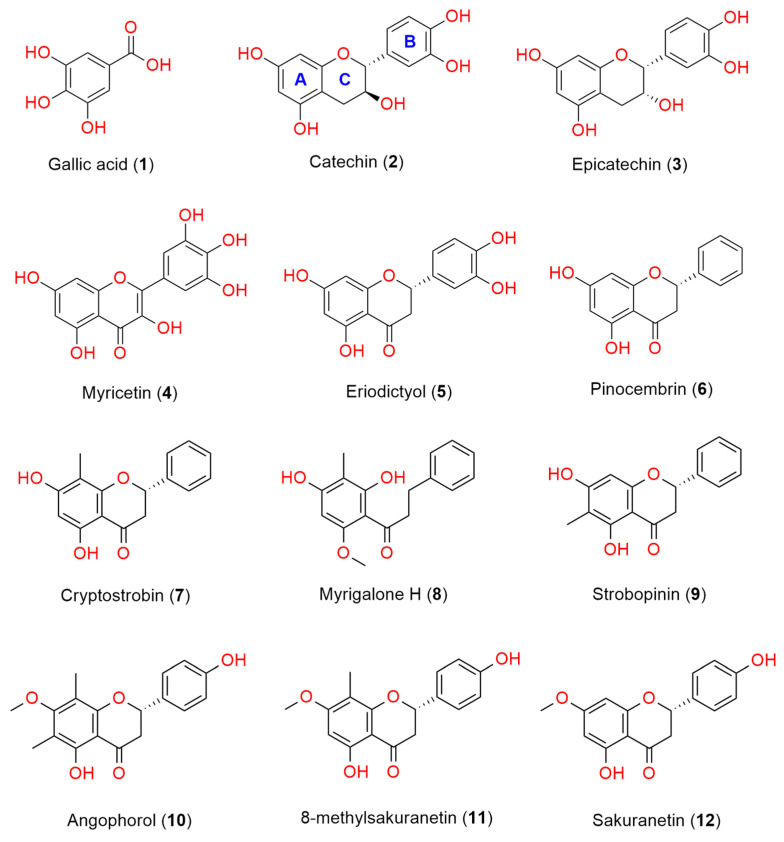
Chemical structures of compounds isolated from the propolis of stingless bees *Tetragonula carbonaria* from Gibberagee, Australia. (A, B and C in blue are labelled for the flavonoid ring system).

The propolis extract exhibited a scavenging property against the free radical DPPH by 80% at a concentration of 100 μg/mL and displayed an IC_50_ value of 24.5 μg/mL (Table 1). Bioassay-guided fractionation was employed to isolate one phenolic acid, gallic acid (**1**) [25], and eleven flavonoids, including catechin (**2**) [26,27], epicatechin (**3**) [26,27], myricetin (**4**) [28], eriodictyol (**5**) [29], pinocembrin (**6**) [30], cryptostrobin (**7**) [12], myrigalone H (**8**) [31], strobopinin (**9**) [12], angophorol (**10**) [32], 8-methylsakuranetin (**11**) [32], and sakuranetin (**12**) [33]. Their chemical structures are depicted in Figure 1 and were verified by comparing their NMR and MS data with previous data reported in the literature.

The DPPH radical scavenging assay was used to evaluate the antioxidant activities of all isolated compounds, as shown in Table 1. Nine of the twelve compounds exhibited greater than 50% inhibition of DPPH radicals at 100 µg/mL, and five of them showed more potent antioxidant activity than L-ascorbic acid (vitamin C) (Table 1). Gallic acid (**1**) demonstrated the most potent antioxidant activity with an IC_50_ value of 0.93 µg/mL. Among the 11 flavonoids tested, the flavonol myricetin (**4**) showed the strongest DPPH radical scavenging activity (IC_50_ of 1.84 µg/mL) and was followed by two flavanols with almost similar antioxidant potency, epicatechin and catechin (IC_50_ of 2.28 and 2.49 µg/mL, respectively). A direct comparison with the positive control, vitamin C, demonstrates that compounds **1**–**5** exhibit more potent DPPH radical scavenging activity, with IC_50_ values ranging from 5.3 to 11.8 µM compared to 28.4 µM for vitamin C. The data of isolated flavonoids indicated that the number and position of the hydroxyl groups in the rings were crucial to maintaining antioxidant activity. The results were consistent with previous studies in which the presence of a catechol group in ring B (compounds **2**–**5**) and a 3-hydroxyl group in a heterocyclic ring C increased radical scavenging activity, and a 2,3-double bond conjugated with the 4-oxo group in the ring C strengthened the antioxidant activity [34]. No significant difference in the antioxidant activity was observed when a methyl group was attached to ring A (compounds **7** and **9**–**11**). The double inhibition of DPPH at 100 µg/mL between a chalcone, myrigalone H (**8**) (80%), and a flavanone, pinocembrin (38%), indicated that opening the ring C facilitated the scavenging potency. Additionally, the release of a resorcinol moiety in ring A potentially leads to the enhancement of the antioxidant activity.

### 3.2. Mining Polyphenolic Compounds Through Molecular Networking Analysis

**Figure 2:**
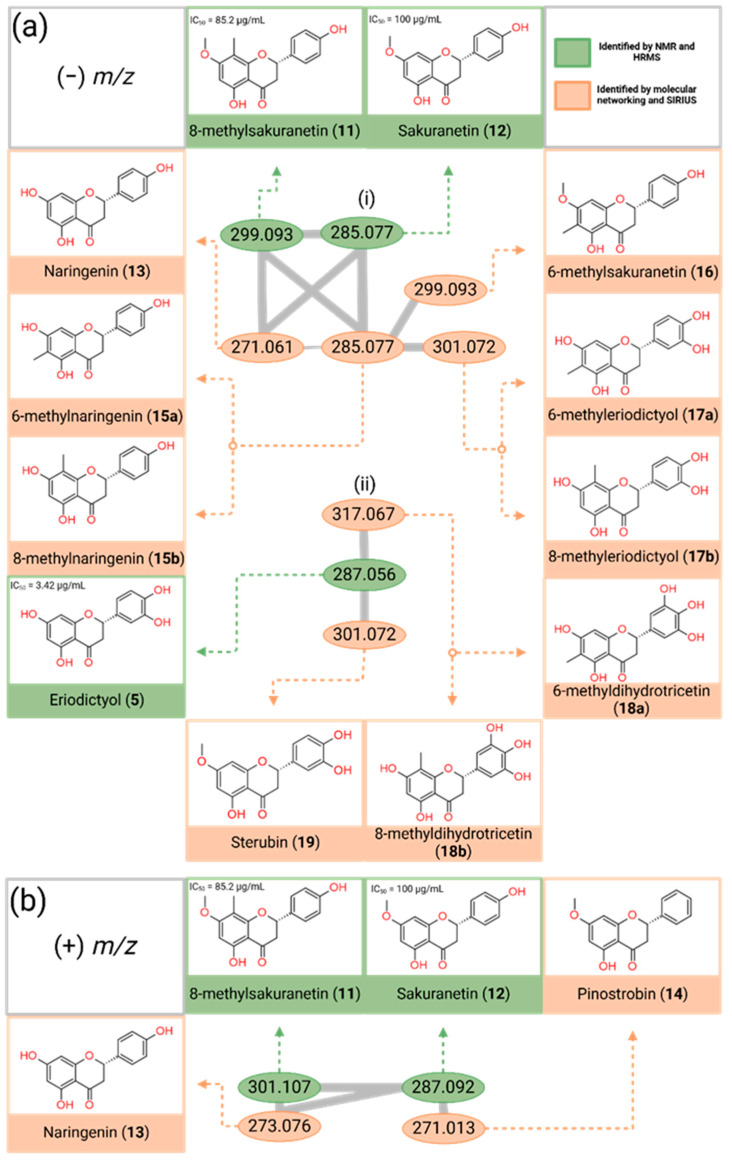
Molecular networking analysis of *T. carbonaria* propolis collected from Gibberagee, Australia, from the negative mode (**a**) and the positive mode (**b**); original network created using GNPS and visualised in Cytoscape, and the network was then recreated in BioRender.com. Green: isolated compounds; orange: tentative compounds. Isolated compounds (Green) have their IC_50_ values that were reported from the DPPH assays performed in this study.

The compound structures depicted in orange in Figure 2 were determined from the elucidation of their MS/MS data and the comparison of their MS/MS data with those of isolated compounds (**1**–**12**). Their structures were then further confirmed by searching the SIRIUS compound database (Table S1 in Supplementary Materials).

Using the negative MS/MS data, GNPS was able to network two clusters of flavonoids containing six (Figure 2a(i)) and three (Figure 2a(ii)) nodes in the negative mode (Figure 2a). Figure 2a(i) further confirmed the presence of 8-methylsakranetin (**11**) ((−) *m*/*z* 299.093) and sakuranetin (**12**) ((−) *m*/*z* 285.077). Four additional flavanones that were not isolated using HPLC were assigned from this molecular networking analysis. These compounds included naringenin (**13**) ((−) *m*/*z* 271.061), 6-methylnaringenin (**15a**) or 8-methylnaringenin (**15b**) ((−) *m*/*z* 285.077), 6-methylsakuranetin (**16**) ((−) *m*/*z* 299.093), and 6-methyleriodicyol (**17a**) or 8-methyleriodicyol (**17b**) ((−) *m*/*z* 301.072). The negative cluster (Figure 2a(ii)) elucidated an additional two compounds, sterubin (**19**) ((−) *m*/*z* 301.072) and 6-methyldihydrotricetin (**18a**) or 8-methyldihydrotricetin (**18b**) ((−) *m*/*z* 317.067).

Using the positive MS/MS data, GNPS created a single cluster of flavonoids (Figure 2b) containing four nodes that were then annotated. Figure 2b further confirmed the presence of compounds 8-methylsakranetin (**11**) ((+) *m*/*z* 301.107) and sakuranetin (**12**) ((+) *m*/*z* 287.092). From this positive cluster, two additional compounds that were not isolated using HPLC were identified, including naringenin (**13**) ((+) *m*/*z* 273.076) and pinostrobin (**14**) ((+) *m*/*z* 271.013).

Based on the compounds determined, it is evident that *T. carbonaria* bees in the Gibberagee region have an affinity for flavonoids that have methyl and methoxy groups on the A-ring. As Australia is considered a megadiverse country, the flora surrounding these bees is likely very different from that in overseas studies. As a result, the native bees of the Gibberagee region collect different plant materials to make their propolis. This distinct difference in the flavonoids may also be attributed to the difference in the bee species enzymes; as they make the propolis, they mix their saliva with the plant material, likely introducing chemical reactions between the saliva and plant material, providing the bees with distinct differences in their propolis composition. *Melichrus gibberagee* and *Eucalyptus punctata* are two Australian endemic plant species that dominate the Gibberagee region [35]. These plants may provide a rich source of flavonoid compounds for native stingless bees to forage, warranting further studies to identify the botanical source of the *T. carbonaria* propolis in Gibberagee.

### 3.3. In Silico Assay

α-glucosidase is one of the most important enzymes in carbohydrate digestion, as this enzyme is primarily responsible for the breakdown of complex carbohydrates, such as oligosaccharides, into monosaccharides. The inhibition of this enzyme leads to the delayed release of glucose into the bloodstream. Drugs such as acarbose and voglibose or nutraceuticals that offer similar effects can be instrumental in lowering postprandial blood sugar levels in those with diabetes mellitus [36].

To understand if compounds identified from Gibberagee propolis have a potential antidiabetic property, they were subjected to an in silico assay against the α-glucosidase enzyme (PDB ID: 2ZQ0). The docked complex is considered the best-docked if it exerts the most negative binding affinity energy value, reflecting a strong protein–ligand interaction and thereby potentially blocking the active site of the enzyme. The binding affinity values and detailed interactions at the binding site of the 19 compounds, along with two positive controls (acarbose and voglibose) towards α-glucosidase, are available in Table S2 (Supplementary Materials). From the docking results, it was found that the positive control, acarbose, exhibited the strongest binding affinity (−10.049 kcal/mol), and, among the identified compounds in this study, 8-methyldihydrotricetin (**18b**) exhibited the strongest binding affinity (−9.578 kcal/mol). The 19 compounds in this study showed affinity energy ranging from −9.578 kcal/mol to −5.981 kcal/mol. All compounds docked in this study showed higher binding affinity towards the enzyme than voglibose, except for gallic acid (**1**) (−7.264 versus −5.981 kcal/mol).

**Figure 3:**
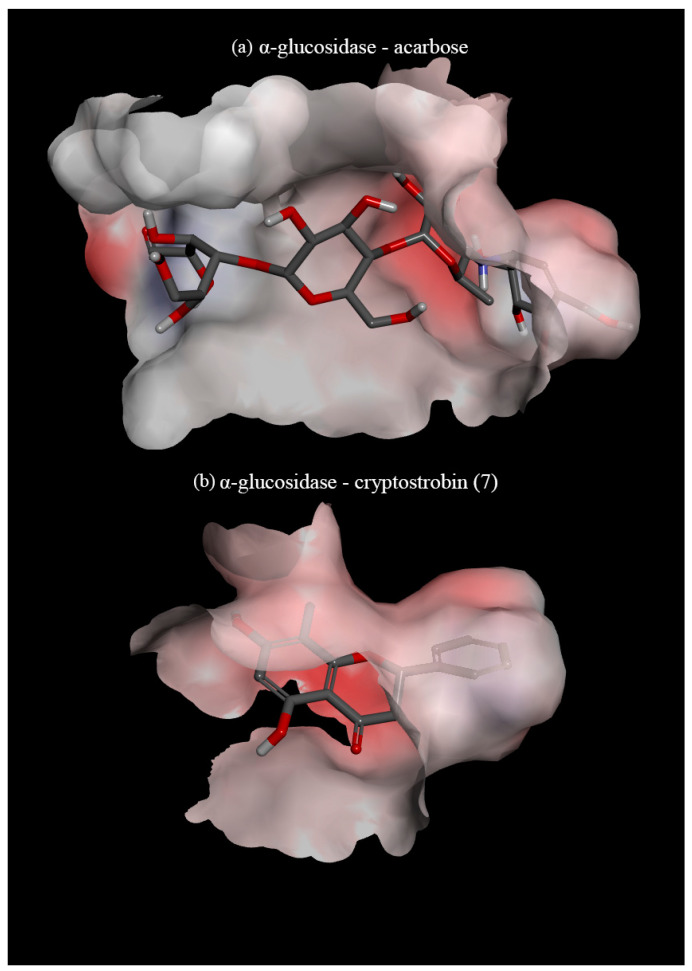
(**a**) Molecular docking of the protein α-glucosidase (2ZQ0) with acarbose (control); (**b**) molecular docking of the protein α-glucosidase (2ZQ0) with cryptostrobin (**7**). Visualised using Discovery Studio.

The docking study indicated that acarbose and voglibose largely depend on hydrogen–hydrogen bonding within the active site of the enzyme (Figure 3a). However, the polyphenolics were found to interact with the active site of the enzyme utilising ring B (Figure 3b), forming electrostatic bonds (π-anion) with the amino acid GLU^439^ and/or GLU^532^. All the polyphenolic compounds in this study formed either π–alkyl or π–π bonds between ring B of the polyphenol and the amino acid VAL^471^ and PHE^536^ (Table S2). These two bonds appear to be the foundation interactions that allow polyphenols to inhibit α-glucosidase. When the compounds have a hydroxy in ring B, this hydroxy creates a hydrogen bond with GLU^391^, enhancing the binding with α-glucosidase. The C-methylation on ring A showed some effects on the binding affinity. C-methylation at position C-8 exhibited a stronger interaction between the polyphenol and the enzyme when compared with the C-methylation at position C-6. This can be seen when comparing compounds **11** (−8.857 kcal/mol) versus **16** (−8.449 kcal/mol), **15b** (−8.275 kcal/mol) versus **15a** (−8.190 kcal/mol), **17b** (−9.224 kcal/mol) versus **17a** (−9.216 kcal/mol), and **18b** (−9.578 kcal/mol) versus **18a** (−9.538 kcal/mol).

The relationship between ring A substitution of flavones and α-glucosidase inhibition was studied extensively in vivo by Gao and his colleagues [37]. It was observed that without any ring B substitutions, ring A hydroxylations at positions C-5, C-6, and C-7 were crucial for α-glucosidase inhibition and that most substitutions at position C-8 reduced the effects of the compound’s ability to inhibit α-glucosidase [37]. However, potential confounding factors between both ring A and ring B hydroxylation, methoxylation, and C-methylation substitutions have not been identified. In our in silico study, an increase in binding affinity with the addition of the C-methylation substitution was observed, as evidenced by the comparison of binding affinity between compounds **11** (−8.857 kcal/mol) and **12** (−8.202 kcal/mol) and between compounds **7** (−8.908 kcal/mol) and **6** (−8.579 kcal/mol).

A previous study also suggested that a pair of hydroxy groups on ring B in positions C-3′ and C-4′ increase the activity of flavonoids in inhibiting α-glucosidase [38]. During the docking of the compounds that were identified, a general trend of the more hydroxy groups on the B ring, the greater the binding activity, was also found, as evidenced by the binding affinity of compounds **17a**, **17b**, **18a**, **18b**, and **19**. This can be further confirmed when comparing the binding affinity of compounds **12** (−8.202 kcal/mol) to **19** (−8.734 kcal/mol), whose difference is the addition of another hydroxy group on ring B at position C-5′. As seen from previous studies on the antioxidant activity of flavonoids, an increase in the hydroxy groups on ring B leads to an increase in free radical scavenging [39,40].

From the results of this molecular docking study, there appears to be a positive relationship between the number of hydroxy groups on ring B, the polyphenols, and the binding affinity of α-glucosidase, further linking the relationship between antioxidant activity and α-glucosidase activity. These findings would encourage further in vitro and in vivo screening to verify the antidiabetic properties of this propolis type and the unique methylated flavonoids present within it.

### 3.4. Potential Bioactivity of Compounds Identified from the Gibberagee Propolis

A literature search on the compounds identified from the Gibberagee propolis demonstrated that many of the compounds exhibited a wide variety of bioactivities, including anti-inflammatory, antioxidant, anticancer, antimicrobial, neuroprotective, antiallergic, anti-angiogenic, anticancer, antiviral, cardioprotective, and antidiabetic properties (Table 2).

The antidiabetic effects of eight polyphenolics (compounds **1–5**, **7**, **13,** and **14**) were previously determined through in vitro assays. Although gallic acid (**1**) did not show a strong interaction with α-glucosidase (−5.981 kcal/mol) in the in silico assay compared to other compounds, previous research reported that this compound significantly improved both the antioxidant status and glucose homeostasis of diabetic mice [45]. Compounds **11**, **15a**, **15b**, **16**, **17a**, **17b**, **18a**, and **18b** have been sparsely studied in the literature regarding their potential bioactivities, and no antidiabetic activity has been previously reported for these compounds. The unique combination of polyphenolics with known and unknown antidiabetic properties in the Gibberagee propolis warrants further investigations of its activity through in vitro and in vivo assays.

## 4. Conclusions

The combination of the bioassay-guided fractionation and the GNPS molecular networking analysis led to the identification of several polyphenol subclasses, including flavanones, flavanols, flavanols, and chalcones, with a unique mixture of C-methylated flavonoids on the A-ring for the Gibberagee propolis. Some of these compounds have not been previously investigated for their bioactivities. Five of the identified compounds exhibited 2- to 5-fold greater free radical scavenging activity than vitamin C, while eleven compounds demonstrated stronger α-glucosidase inhibition. The identification of the antioxidant and α-glucosidase inhibitory properties of polyphenolic compounds in this propolis highlights its potential for the development of nutraceutical and cosmeceutical applications, inspiring new avenues of research and product development for this unique propolis. Further in vitro and in vivo studies will be required to validate its therapeutic properties. Additionally, the identification of the botanical sources for this propolis by examining unique flora in the Gibberagee region, such as *Melichrus gibberagee* and *Eucalyptus punctata*, will contribute to the conservation of Australian native plant species.

## Figures and Tables

**Table 1:** Antioxidant activity of isolated compounds in *T. carbonaria* propolis from Gibberagee, Australia.

Extract/Compounds	% Inhibition at 100 µg/mL	IC_50_ (µg/mL)	IC_50_ (µM)
Propolis extract	80 ± 2	24.5 ± 0.3	-
Gallic acid (**1**)	100 ± 1	0.9 ± 0.1	5.3 ± 0.6
Catechin (**2**)	100 ± 0	2.5 ± 0.2	8.6 ± 0.7
Epicatechin (**3**)	99 ± 1	2.3 ± 0.1	7.9 ± 0.3
Myricetin (**4**)	100 ± 1	1.8 ± 0.1	5.7 ± 0.3
Eriodictyol (**5**)	100 ± 0	3.4 ± 0.2	11.8 ± 0.7
Pinocembrin (**6**)	38 ± 3	N.D ^a^	N.D ^a^
Cryptostrobin (**7**)	41 ± 5	N.D ^a^	N.D ^a^
Myrigalone H (**8**)	80 ± 2	20.0 ± 0.5	69.9 ± 1.7
Strobopinin (**9**)	40 ± 1	N.D ^a^	N.D ^a^
Angophorol (**10**)	63 ± 4	58.4 ± 0.8	186 ± 2.5
8-Methylsakuranetin (**11**)	56 ± 4	85.2 ± 1.1	284 ± 3.7
Sakuranetin (**12**)	50 ± 1	100 ± 0.1	350 ± 0.3
Ascorbic acid (Vitamin C)	100 ± 0	5.0 ± 0.1	28.4 ± 0.6

N.D ^a^: Not determined due to lower 50% inhibition against DPPH at 100 µg/mL, which was the maximum concentration tested.

**Table 2:** Known bioactivities of compounds identified in *T. carbonaria* propolis from Gibberagee, Australia, as reported in the literature.

Compound	Known Bioactivities
Gallic Acid (**1**)	Antiallergic [41], Anti-angiogenic [42], Anticancer [43], Antidiabetic [44,45], Anti-inflammatory [46], Antimicrobial [43,44], Antioxidant [44], Antiviral [44], Neuroprotective [44]
Catechin (**2**)	Cardiovascular Protective [47], Anticancer [48], Antidiabetic [49], Anti-inflammatory [50], Antimicrobial [51], Antioxidant [47], Neuroprotective [47]
Epicatechin (**3**)	Anticancer [47], Antidiabetic [49,52], Antioxidant [47], Cardiovascular protective [47], Neuroprotective [52]
Myricetin (**4**)	Anticancer [53], Antidiabetic [53], Antihypertensive [53], Antimicrobial [53], Antioxidant [53], Immunomodulatory [53], Neuroprotective [53]
Eriodictyol (**5**)	Anticancer [54], Antidiabetic [54], Anti-inflammatory [54], Antioxidant [54], Cardioprotective [54], Hepatoprotective [54], Neuroprotective [54]
Pinocembrin (**6**)	Anticancer [55], Antifibrotic [55], Anti-inflammatory [55], Antimicrobial [55], Antioxidant [55], Cardiovascular protective [55], Neuroprotective [55]
Cryptostrobin (**7**)	Antibacterial [12,56], Antidiabetic [57], Antihypertensive [56]
Myrigalone H (**8**)	Antibacterial [58]
Strobopinin (**9**)	Anti-inflammatory [59], Antimicrobial [59], Antioxidant [59], Antiparasitic [59], Neuroprotective [60]
Angophorol (**10**)	Anticancer [61]
Sakuranetin (**12**)	Antiallergic [62], Anticancer [62], Anti-inflammatory [62], Antimicrobial [62], Antimutagenic [62], Antioxidant [62], Antiparasitic [62], Antiviral [62]
Naringenin (**13**)	Anticancer [63], Antidiabetic [63], Antimicrobial [63], Antidiabetic [63], Antioxidant [63], Cardiovascular protective [63], Gastroprotective [63], Immunomodulatory [63], Neuroprotective [63]
Pinostrobin (**14**)	Antibacterial [64], Anticancer [64,65], Antidiabetic [65], Anti-inflammatory [65], Antioxidant [64], Antiviral [64]
Sterubin (**19**)	Anti-inflammatory [66], Antioxidant [66], Neuroprotective [66]

## Data Availability

The original contributions presented in this study are included in the article/Supplementary Materials. The original GNPS data can be found at the following links: Negative MS/MS data: https://gnps.ucsd.edu/ProteoSAFe/status.jsp?task=ee158423f09a4993b166ac837c5d4204 (accessed on 15 August 2024). Positive MS/MS data: https://gnps.ucsd.edu/ProteoSAFe/status.jsp?task=ee158423f09a4993b166ac837c5d4204 (accessed on 15 August 2024). Further inquiries can be directed to the corresponding author.

## Supplementary Materials

The following supporting information can be downloaded at https://www.mdpi.com/article/10.3390/foods14060965/s1, Table S1: Description of compounds identified from Gibberagee stingless bee propolis using a combination of GNPS and SIRIUS from negative and positive mode MS/MS data; Table S2: Binding affinity and binding interaction of compounds **1–19** to α-glucosidase (2ZQ0); Figure S1: Global Natural Products Social Molecular Networking non-clustered compounds visualised in Cytoscape (version 3.10.3); Figure S2: Non-covalent interactions of sakuranetin (**12**) and acarbose (control) with *α*-glucosidase (2ZQ0); Figure S3: Comparisons between the MS^1^ spectra of some identified compounds and those in the SIRUS databases.
