# Supplementary material for: Bioactive Polyphenolic Compounds from Propolis of Tetragonula carbonaria in the Gibberagee Region, New South Wales, Australia
Source: Foods. 2025 Mar 12;14(6):965. doi: 10.3390/foods14060965 (PMC11941244; doi:10.3390/foods14060965)
Supplement: Supplementary file 1 [file foods-14-00965-s001.zip › foods-3495165-supplementary.pdf]

Supplementary Material

Bioactive Polyphenolic Compounds from Propolis of *Tetragonula carbonaria* in the Gibberagee Region, New South Wales, Australia

Dylan W. Ebner [1,2], Damon C. Woods [1,2] and Trong D. Tran [1,2,*]

[1] Centre for Bioinnovation, University of the Sunshine Coast, Sippy Downs, QLD 4556, Australia
[2] School of Science, Technology and Engineering, University of the Sunshine Coast, Sippy Downs, QLD 4556, Australia
* Correspondence: ttran1@usc.edu.au; Tel.: +61-7-5459-4579

Contents of Supplementary Material

No.	Contents
1	**Table S1.** Description of compounds identified from Gibberagee stingless bee propolis using a combination of GNPS and SIRIUS from negative and positive mode MS/MS data.
2	**Table S2.** Binding affinity and binding interactions of compounds **1-19** to α-glucosidase (2ZQ0)
3	**Figure S1.** Global Natural Products Social Molecular Networking non-clustered compounds visualised in Cytoscape (version 3.10.3).
4	**Figure S2.** Non-covalent interactions of sakuranetin (**12**) and acarbose (control) with α-glucosidase (2ZQ0)
5	**Figure S3.** Comparisons between the MS spectra of some identified compounds and those in the SIRUS databases

 https://doi.org/10.3390/foods14060965

Table S1. Description of compounds identified from Gibberagee stingless bee propolis using a combination of GNPS and SIRIUS from negative and positive mode MS/MS data.

Compound Name	Figure location	Retention Time (Min)	Calculated Precursor Mass [M-H]- / [M+H]+	Precursor Mass (Sirius) [M-H]- / [M+H]+	Precursor Mass (GNPS) [M-H]- / [M+H]+	Sirius Confidence (%)
Gallic Acid (**1**)	S2	1.02	169.0142	N.D	169.014	N.D
Catechin (**2**)	S2	3.96	289.071	289.071	289.072	99.55
Epicatechin (**3**)	S2	2.38	289.071	289.071	289.072	99.55
Myricetin (**4**)	N.D	N.D	319.046	N.D	N.D[a]	N.D[a]
Eriodictyol (**5**)	A (-), (ii)	3.64	287.056	N.D	287.056	N.D[a]
Pinocembrin (**6**)	N.D	N.D	255.066	N.D	N.D[a]	N.D[a]
Cryptostrobin (**7**)	S2	4.34	269.081	269.081	269.082	93.57
Myrigalone H (**8**)	S2	2.80	285.113	285.113	285.114	87.83
Strobopinin (**9**)	N.D	N.D	283.097	283.0966	269.082	81.61
Angophorol (**10**)	N.D	6.44	313.108147	313.1083	N.D[a]	62.82
8-Methylsakuranetin (**11**)	A (-), (i); B (+)	5.30	299.092497 / 301.10705	299.0932 / 301.1078	299.093 / 271.013	81.22 / 85.56
Sakuranetin (**12**)	A (-), (i); B (+)	4.78	285.076847	285.0763	285.077	84.98
Naringenin (**13**)	A (-), (i); B (+)	4.48	271.061197	271.06174	271.061	100
Pinostrobin (**14**)	B (+)	8.02	269.081932/ 271.096485	269.0823	N.D[a]	81.22
6-Methylnaringenin (**15a**)	A (-), (i)	5.27	285.076847	285.0770	285.077	85.77
8-Methylnaringenin (**15b**)	A (-), (i)	5.27	285.076847	285.0770	285.077	84.62
6-Methylsakuranetin (**16**)	A (-), (i)	6.21	299.092497	299.0932	299.093	97.58
6-Methyleriodictyol (**17a**)	A (-), (i)	4.96	301.071762	301.0720	301.072	88.24
8-Methyleriodictyol (**17b**)	A (-), (i)	4.96	301.071762	301.0720	301.072	N.D[a]
6-Methyldihydrotricetin (**18a**)	A (-), (ii)	3.68	317.066676	317.0669	317.067	72.27
8-Methyldihydrotricetin (**18b**)	A (-), (ii)	3.68	317.066676	317.0669	317.067	72.27
Sterubin (**19**)	A (-), (ii)	4.99	301.071762	301.072	301.072	100

N.D[a]: Not determined or not found using the MS/MS data mining software used throughout this study.

 https://doi.org/10.3390/foods14060965

Table S2. Binding affinity and binding interactions of compounds **1-19** to α-glucosidase (2ZQ0)

Compound Name	Binding affinity (kcal/mol)	Hydrogen bond (Distance, Å)	Hydrophobic bond (Distance, Å)	Electrostatic bond (Distance, Å)
Acarbose	-10.049	Ser^{217} (2.566); His^{507} (1.918); Arg^{529} (2.506); Glu^{532} (2.932); Glu^{532} (2.809); Glu^{391} (2.868); Glu^{508} (2.809); Glu^{526} (2.449); Glu^{194} (2.381); Glu^{508} (2.500); Phe^{536} (2.332)	-	-
Voglibose	-7.264	Lys^{467} (1.794); His^{507} (2.014); Glu^{194} (2.657); Tyr^{533} (2.324); Glu^{532} (2.031)	-	-
Gallic Acid (**1**)	-5.981	His^{507} (2.028); Glu^{526} (2.478); Glu^{532} (2.479)	**π-alkyl**: Val^{471} (5.101)	**π-anion:** Glu^{508} (4.946)
Catechin (**2**)	-8.638	Asn^{216} (2.687); Glu^{391} (1.731); Gly^{469} (3.745); Glu^{532} (3.574)	**π-alkyl:** Val^{471} (4.902)	**π-anion:** Glu^{439} (4.039); Glu^{532} (4.689); Glu^{532} (4.504)
Epicatechin (**3**)	-8.868	Asn^{216} (2.462); His^{437} (2.342); Glu^{391} (2.218)	**π-π:** Phe^{536} (5.576); Phe^{401} (5.929); Try^{533} (5.883) **π-alkyl:** Val^{471} (5.184); Val^{471} (5.358)	**π-anion:** Glu^{439} (3.799)
Myricetin (**4**)	-8.753	Trp^{331} (1.962); Lys^{467} (2.959); Glu^{439} (2.948); Glu^{391} (1.833); Glu^{391} (2.761); Glu^{508} (2.505)	**π-π:** Phe^{401} (4.937) **π-alkyl:** Val^{471} (5.037)	**π-anion:** Glu^{532} (4.031)
Eriodictyol (**5**)	-8.893	Lys^{467} (2.780)	**π-π:** Phe^{536} (5.427); Try^{533} (5.982) **π-alkyl:** Val^{471} (5.440)	**π-anion:** Glu^{439} (4.289); Glu^{532} (4.348)
Pinocembrin (**6**)	-8.579	Ser^{217} (2.159)	**π-π:** Phe^{536} (5.438); Tyr^{533} (5.995) **π-alkyl:** Val^{471} (5.076)	**π-anion:** Glu^{439} (3.667)
Cryptostrobin (**7**)	-8.908	Ser^{217} (2.605); Ser^{217} (1.958); Glu^{194} (2.379)	**π-π:** Phe^{536} (5.542); Phe^{401} (5.957) **π-alkyl:** Val^{471} (4.998); Val^{471} (5.417)	**π-anion:** Glu^{439} (3.455)
Myrigalone H (**8**)	-7.981	-	**π-π:** Phe^{536} (3.907) **π-alkyl:** Val^{471} (5.022)	-
Strobopinin (**9**)	-8.904	Asn^{216} (2.944)	**π-π:** Phe^{536} (5.486) **π-alkyl:** Val^{471} (5.033)	**π-anion:** Glu^{439} (3.598)
Angophorol (**10**)	-8.707	His^{437} (3.091); Glu^{391} (2.212); Try^{533} (2.247)	**π-π:** Phe^{536} (4.232) **π-alkyl:** Phe^{536} (4.608); Val^{471} (5.496)	**π-anion:** Glu^{439} (4.190)
8-Methylsakuranetin (**11**)	-8.857	Glu^{508} (1.960); Glu^{194} (2.823); Tyr^{533} (3.002); Met^{334} (3.613)	**π-π:** Trp^{341} (4.739) **π-alkyl:** Val^{471} (4.385)	-
Sakuranetin (**12**)	-8.202	Ser^{217} (2.621); Ser^{217} (2.184); Glu^{439} (2.340)	**π-σ:** Tyr^{533} (5.840) **π-π:** Val^{471} (5.452) **π-alkyl:** Phe^{536} (3.714)	**π-anion:** Glu^{439} (4.677); Glu^{532} (3.813);
Naringenin (**13**)	-8.383	His^{507} (2.840); Glu^{508} (1.901);	**π-π:** Phe^{536} (4.259) **π-alkyl:** Val^{471} (5.244)	**π-anion:** Glu^{439} (3.980)
Pinostrobin (**14**)	-8.136	Glu^{194} (2.938); Glu^{532} (2.263)	**π-π:** Trp^{341} (4.813) **π-alkyl:** His^{437} (5.117); His^{507} (4.648); Val^{471} (4.992)	**π-anion:** Glu^{439} (4.969); Glu^{532} (3.773)

6-Methylnaringenin (**15a**)	-8.190	Glu^{508} (2.212); Pro^{215} (2.792)	**π-π:** Phe^{536} (4.914)<br>**π-alkyl:** Val^{471} (4.690)	**π-anion:** Glu^{532} (3.876)
8-Methylnaringenin (**15b**)	-8.275	Asn^{216} (2.963); Ser^{217} (2.692); Ser^{217} (1.965); Glu^{194} (2.565)	**π-π:** Phe^{536} (4.123); Phe^{536} (4.904)	-
6-Methylsakuranetin (**16**)	-8.449	Ser^{217} (2.532); Ser^{217} (2.133); Glu^{391} (2.151)	**π-π:** Tyr^{533} (5.842)<br>**π-alkyl:** Val^{471} (5.346)	**π-anion:**Glu^{439} (4.660); Glu^{532} (3.867)<br>**π-σ:** Phe^{536} (3.593)
6-Methyleriodictyol (**17a**)	-9.216	Ser^{217} (1.924); Glu^{391} (2.822); Glu^{508} (2.347); His^{437} (3.565)	**π-alkyl:** Val^{471} (5.396)	**π-anion:** Glu^{532} (4.244)
8-Methyleriodictyol (**17b**)	-9.224	Lys^{467} (2.485); Glu^{391} (1.879)	**π-π:** Trp^{341} (5.603); Phe^{536} (5.513); Phe^{401} (5.847)<br>**π-alkyl:** Val^{471} (5.175)	**π-anion:** Glu^{439} (4.090)
6-Methyldihydrotri-cetin (**18a**)	-9.538	Lys^{467} (2.852); Glu^{391} (1.945)	**π- alkyl:** Val^{471} (5.230)	**π-anion:** Glu^{439} (4.195)
8-Methyldihydrotri-cetin (**18b**)	-9.578	Ser^{217} (2.192); Ser^{217} (1.929); Trp^{341} (2.803); Glu^{391} (2.344); Glu^{508} (2.945); Glu^{194} (2.862); His^{437} (2.741); Lys^{467} (3.711)	**π-π:** Phe^{536} (5.398); Phe^{401} (5.740)<br>**π-alkyl:** Val^{471} (5.296); Val^{471} (5.402)	**π-anion:** Glu^{439} (3.791)
Sterubin (**19**)	-8.734	His^{507} (2.732); Glu^{508} (1.986); Glu^{526} (2.255)	**π-σ:** Val^{471} (3.948); Phe^{536} (4.135)	**π-anion:** Glu^{508} (4.733)

Figure S1. Global Natural Products Social Molecular Networking non-clustered compounds visualised in Cytoscape (version 3.10.3) in the negative mode.

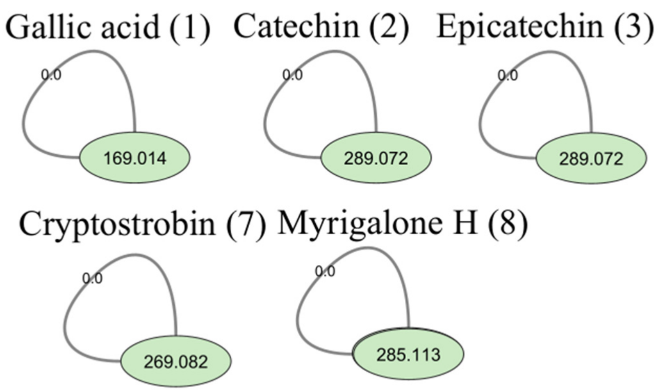

Figure S2. Non-covalent interactions of sakuranetin (**12**) and acarbose (control) with α-glucosidase (2ZQ0)

A)

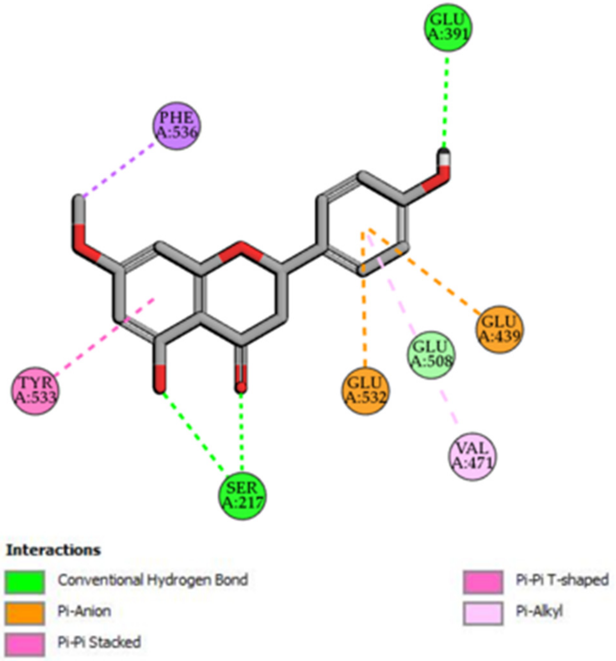

B)

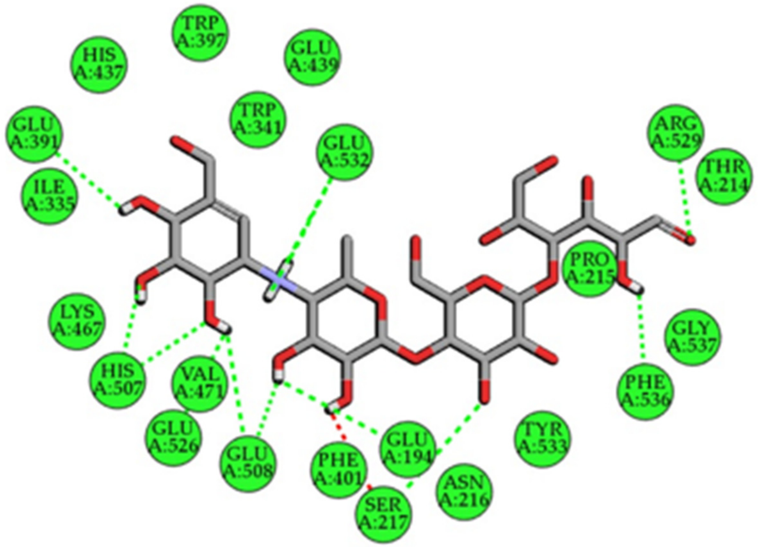

Figure S3. Comparisons between the MS spectra of some identified compounds and those in the SIRUS databases

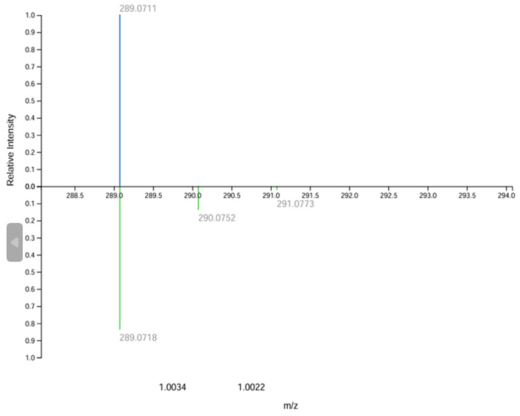

Catechin (**2**)

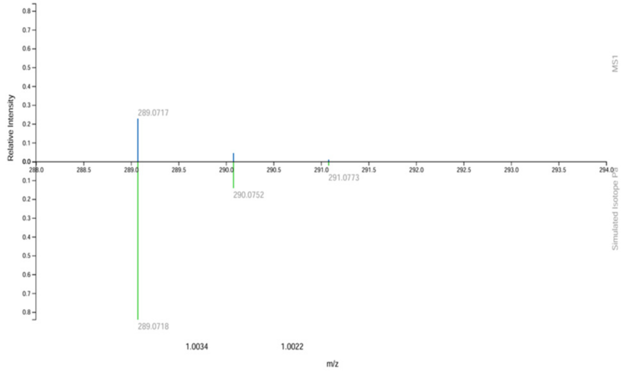

Epicatechin (**3**)

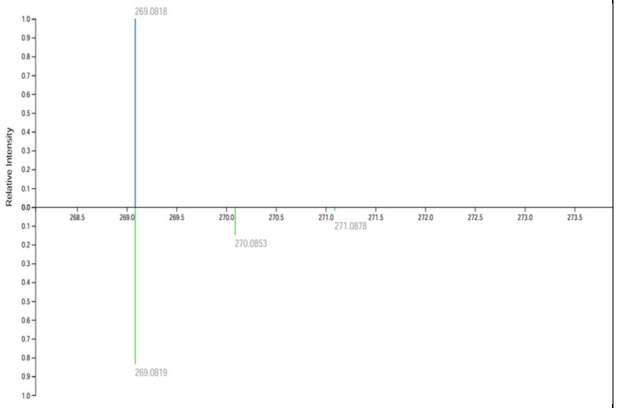

Cryptostrobin (**7**)

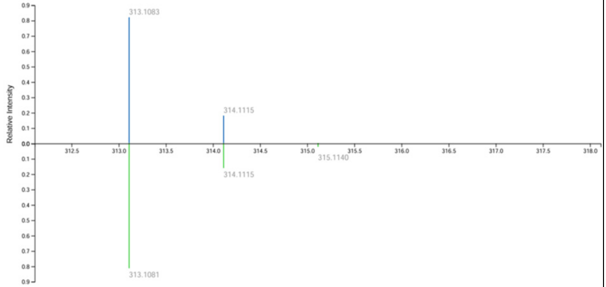

Angophorol (**10**)

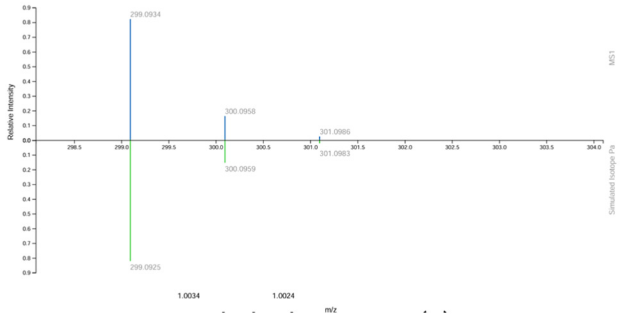

8-Methylsakuranetin (**11**)

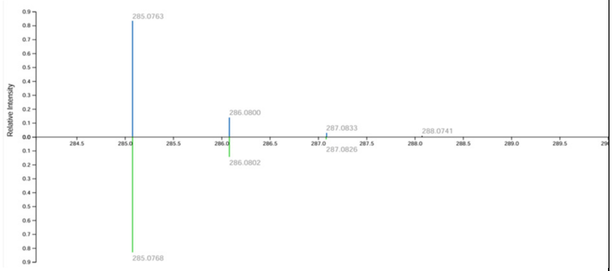

Sakuranetin (**12**)

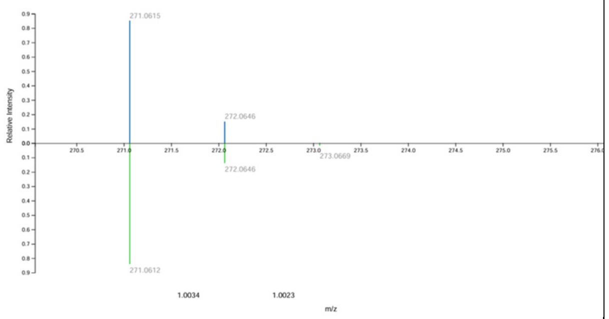

Naringenin (**13**)

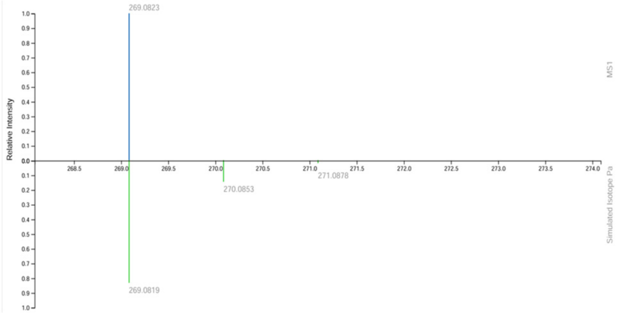

Pinostrobin (**14**)

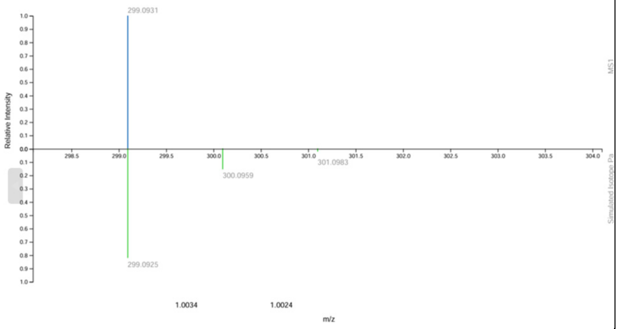

6-Methylsakuranetin (**16**)

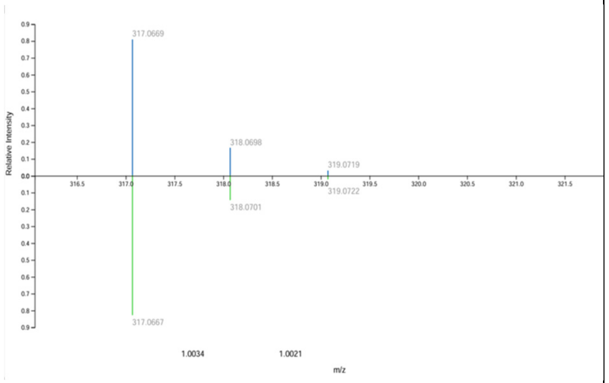

6-Methyleriodictyol (**17a**) or 8-Methyleriodictyol (**17b**)

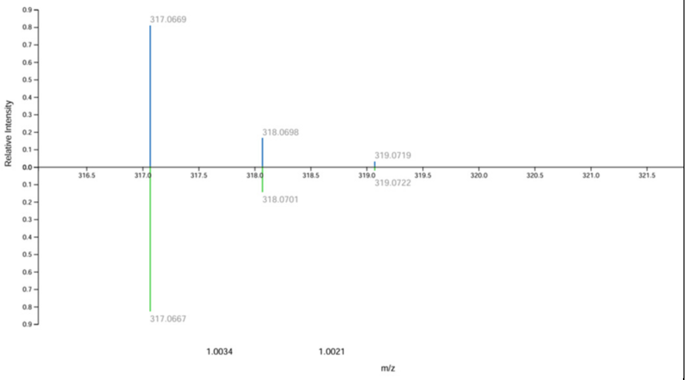

6-Methyldihydrotricetin (**18a**) or 8-Methyldihydrotricetin (**18b**)

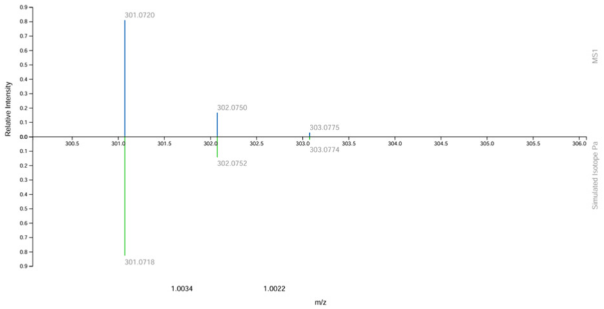

Sterubin (**19**)
